# The Evolution of Accelerated Diagnostic Protocols for Suspected Myocardial Infarction

**DOI:** 10.3390/jcm15135125

**Published:** 2026-07-01

**Authors:** James Hatherley, Paul Collinson, Tarek Abuzahra, Aleem Khand

**Affiliations:** 1 Department of Cardiology, Liverpool University Hospital NHS Foundation Trust, Liverpool L7 8YE, UKtarek.abuzahra@liverpoolft.nhs.uk (T.A.); 2 Institute of Life Courses and Medical Sciences, University of Liverpool, Liverpool L69 3GL, UK; 3 City St Georges, University of London, London EC1V 0HB, UK; paul.collinson@ntlworld.com; 4 Department of Cardiology, Liverpool Heart and Chest Hospital, Liverpool L14 3PE, UK

**Keywords:** accelerated diagnostic pathways, troponin, cardiac biomarkers, myocardial infarction, acute coronary syndrome

## Abstract

There have been considerable developments in the analytic precision of cardiac troponins in the last three decades. Whilst there has been near-universal uptake of this technology, there is considerable variability in how to assess acute chest pain patients using high-sensitivity cardiac troponins. This review describes the historical narrative for cardiac troponins and details the evidence base behind decision rules, such as single sample rule-out, single sample rule-in, and accelerated diagnostic protocols (ADPs). There is particular focus on the European Society of Cardiology (ESC) 0/1 and 0/3 h and the high-STEACS ADPs. The ESC 0/3 h ADP appears to have reduced rule-out safety compared to both the ESC 0/1 h and high-STEACS ADP. However, whilst high-STEACS performed well in its validation population, external validation in the US has been less impressive and warrants further investigation. The ESC 0/1 h pathway has demonstrated strong rule-out performance, helped by its observational zone. However, real world implementation studies comparing these ADPs are required to understand their impact on Emergency Department efficiency and the safety of clinician decision-making.

## 1. Methods

This review was conducted following a literature search and review in 2023, conducted on the PubMed database. It did not follow a strict systematic review methodology. Instead, the following search terms were used: ‘troponin’, ‘accelerated diagnostic pathway’, ‘myocardial infarction’, ‘acute coronary syndrome’, ‘single’, ‘European Society of Cardiology or ESC’, ‘1 or one’, ‘3 or three’ and ‘high-STEACS’. Articles were prioritised from meta-analyses to randomised controlled trials and then observational studies. Any relevant articles included in published meta-analyses not found using the search strategy were later included.

## 2. Development of the Accelerated Diagnostic Pathway with the Advent of High-Sensitivity Troponins

The first radioimmunoassay for cTn (cardiac troponin) was developed in the late 1990s [1]. The cardiac specificity of this assay was an advance on other assays of myocyte necrosis such as creatine kinase, lactate dehydrogenase or aspartate transaminase [1,2].

Contemporary cTn assays at this time had inferior diagnostic performance to current hs-cTn assays. The limit of detection (LoD) for first-generation troponin T assays was 50 ng/L and the 10% limit of quantification (LoQ) was 270 ng/L [3]. This is in marked contrast to current hs-cTnT assays that have an LoD of 3 ng/L and a 10% LoQ of 5.4 ng/L [4].

Despite these flaws, contemporary cTn assays improved not only the assessment of patients with suspected MI but also the way it was defined [5,6]. A study published in the New England Journal of Medicine demonstrated the superiority of the cTn assay for the diagnosis of myocardial infarction (MI) over the current gold-standard biomarker of the time, CK-MB [7].

These findings led to the widespread roll-out of cTn and the development of the first universal definition of MI in a consensus paper [5]. This stated that a rise or fall in cardiac biomarkers, preferentially cTn, was essential in the diagnosis of MI [5]. However, up to this point, there was a lack of clinical guidance on how to implement and use cTn. Therefore, clinical practice was inconsistent across hospital trusts.

**Figure 1:**
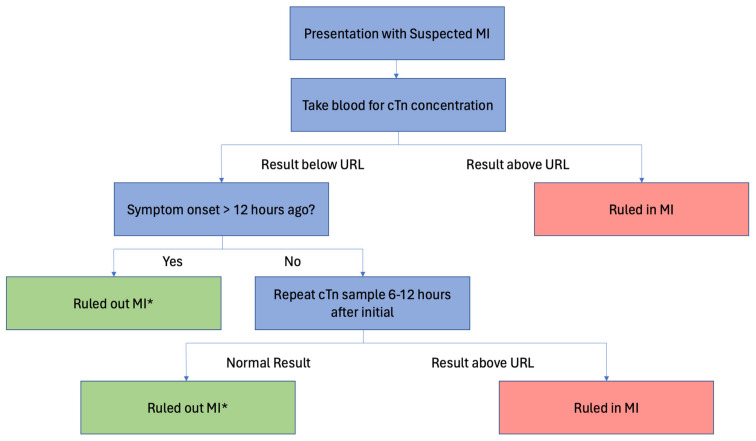
Rudimentary diagnostic pathway constructed from 2008 ESC NSTEMI guidelines. * Patients could only be ruled out if accompanying risk score, GRACE or TIMI, were also low. MI indicates myocardial infarction; cTn, cardiac troponin; GRACE, global registry of acute coronary events; TIMI, thrombolysis in myocardial infarction and URL, 99th percentile upper reference limit.

In their guidelines on Non ST-Elevation MI (NSTEMI) in 2008, the European Society of Cardiology (ESC) provided advice on how to triage patients with suspected NSTEMI using a contemporary cTn assay [8]. A visual representation of this guidance is shown in Figure 1.

Between the ESC 2008 and 2015 guidelines there were rapid improvements in the analytical and diagnostic performance of cTn assays [8,9]. This led to the terms ‘sensitive’ and ‘high-sensitivity’ to describe cTn assays dependent on their analytical performance. High-sensitivity cTn (hs-cTn) assays were commercially available from 2010 onwards; the definitions of these assays are shown in Table 1 [10,11,12,13,14,15,16,17,18].

Subsequently, multiple observational studies confirmed the superior performance of sensitive or hs-cTn assays (Table 2) [10,11,12,13,14]. The most consistent and significant finding was that higher sensitivity led to earlier diagnosis and rule-out of MI. However, by 6 hours the clinical performance was similar between contemporary and hs-cTn assays [12].

Despite the growing body of evidence surrounding hs-cTn, there was still considerable clinician uncertainty about how best to utilise hs-cTns as a new diagnostic resource [19]. This uncertainty led to marked variation in practice. Admission rates and duration of stay varied considerably across hospital trusts.

All subsequently developed accelerated diagnostic pathways (ADPs) stress the importance of interpreting hs-cTn results in the context of the clinical presentation, ECG findings and pre-test probability of MI.

**Table 2:** Summary of original research articles comparing contemporary to ‘sensitive’ or ‘high-sensitivity’ assays, prior to first ESC accelerated diagnostic pathway published in 2015 guidelines.

Article	Assays Compared	Study Pop (Total)	Prevalence of MI (%)	Study Design	Main Findings
Comparison Studies					
Keller et al. [10]	s-cTn and c-cTn	1688	283 (17)	Prospective Observational	At presentation s-cTn was superior to c-cTnI in Dx of MI
Giannitsis et al. [12]	hs-cTn and c-cTn	57	26 (46)	Prospective Observational	At presentation hs-cTnT was superior to c-cTnT for Dx of MI. Equivalent when samples taken 6 h later.
Reichlin et al. [11]	Four s-cTn and one c-cTn	718	123 (17)	Prospective Observational	At presentation all s-cTn were superior to c-cTnT for Dx of MI.
Keller et al. [13]	hs-cTn and c-cTn	1818	413 (22.7)	Prospective Observational	At presentation hs-cTnI was superior to c-cTnI for Dx of MI. Equivalent when samples taken 3 h later.
Weber et al. [14]	hs-cTn and c-cTn	1023	826 (81)	Prospective Observational	At presentation hs-cTnT was superior to c-cTnT for Dx of MI.
Early ADP Validation					
Reichlin et al. [17]	hs-cTnT	872	147 (17)	Prospective Observational	0/1 h ADP had 100% NPV and sensitivity for rule-out of MI; 23% in observe with 8% MI rate in observe.
Than et al. [15]	c-cTnI	1975	302 (15.3)	Prospective Observational	ADP = two negative c-cTnI at 0 + 2 h, normal ECG and TIMI = 0. 20% of patients ruled out, sensitivity 99.7% and NPV 99.7% for 30-day MACE.
Haaf et al. [16]	hs-cTn	887	127 (14)	Prospective, Observational	MI had higher presentation hs-cTn than acute cardiac non-coronary disease, with a higher delta over 1 h.
Rubini Gimenez et al. [18]	hs-cTn	hs-cTnT (Roche)—2072 hs-cTnI (Siemens)—1180 hs-cTnI (Beckman)—1151 hs-cTnI (Abbott)—1567	443 (21) 235 (20) 216 (19) 310 (20)	Prospective Observational	Undetectable hs-TnT at presentation had sensitivity of 98.2% and NPV 98.6% and could rule out 26.5%. All hs-TnI assay results similar but hs-TnT could rule out a greater %.
Rubini Gimenez et al. [20]	hs-cTnI	1811 906 Derivation 905 Validation	329 (18) 161 (18) 168 (19)	Prospective Observational Derivation and Validation.	In both cohorts sensitivity and NPV of rule-out over 97% and 99%, respectively; with PPV in rule-in over 73%; 25–30% of patients in observation zone.
Reichlin et al. [21]	hs-cTnT	1320	229 (17)	Prospective Observational Validation.	Sensitivity and NPV of rule-out was 99.6% and 99.9%, respectively; PPV for rule-in was 78.2%; 24.1% of patients in observation zone.

MI indicates myocardial infarction; s-cTn, sensitive cardiac troponin; c-cTn contemporary cardiac troponin; hs-cTn, high-sensitivity cardiac troponin; Dx, diagnosis; ADP, accelerated diagnostic pathway; NPV, negative predictive value; ECG, electrocardiogram and TIMI, thrombolysis in myocardial infarction score.

## 3. Accelerated Diagnostic Pathways Using a Single hs-cTn Result

Several accelerated diagnostic pathways (ADPs) utilise single sample rule-out (SSRO) to identify low-risk patients whose MI can be ruled out after one hs-cTn result and a non-ischaemic ECG [22].

The evidence supporting the safety of single sample rule-out for both hs-cTnT and TnI is strong and extensive [23,24,25,26,27,28].

It was first evaluated in 2011 by a prospective observational study of 703 patients using hs-cTnT [29]. It demonstrated a sensitivity and negative predictive value (NPV) for rule-out, using the limit of blank (LoB) as the SSRO threshold, of 100% [29]. This was changed to the LoD rather than LoB due to concerns regarding the high level of analytical imprecision at the LoB. Following this paper, many studies, mostly observational except for one randomised controlled trial (RCT), have evaluated the safety of SSRO using the LoD or another optimised value (Table 3).

The proportion of patients eligible for discharge using the LoD appears to be higher with hs-cTnT compared to hs-cTnI assays [23,25,26,30,31,32,33]. This is excluding one study investigating the Architect hs-cTnI assay, which was retrospective and had a very low prevalence of MI [23]. The proportion suitable for discharge using the LoD on the hs-cTnI assay appears to be around 20–30%. However, when the SSRO is increased above the hs-cTnI assays’ LoD, the proportion suitable for discharge is similar to hs-cTnT [24,34,35]. However, doing so can impair clinical performance and safety. Therefore, further studies are required to further validate thresholds above the LoD using hs-cTnI [24,34].

Overall, SSRO using the LoD for both hs-cTnT and I is validated and appears safe. Clinical utility is greatest using hs-cTnT, and further research is required to validate SSRO thresholds above the hs-cTn assay LoD.

**Table 3:** Summary of evidence for single sample rule-out using hs-cTnT and TnI.

Article	Assay	SSRO Threshold	Proportion Ruled Out (%)	Study Design	Primary Outcome	Population Size (n)	Primary Outcome Prevalence (%)	Sensitivity (95% CI)	NPV (95% CI)
High-Sensitivity Cardiac Troponin T									
Body et al. (2011) [29]	Roche, Elecsys	<3 ng/L	27.7	Prospective Observational	Index MI	703	130 (18.5)	100% (97.2–100%)	100% (98.1–100%)
Carlton et al. (2020) [25]	Roche, Elecsys	<5 ng/L	45	Randomised Controlled Trial	MACE at 30 days	316	25 (8)	100%	100%
Sandoval et al. (2022) [36]	Roche, Elecsys	<6 ng/L	29	Prospective Observational	Index MI	1979	141 (7.1)	99.3% (96.1–100%)	99.8% (99.1–100%)
Body et al. (2015) [26]	Roche, Elecsys	<5 ng/L	49.5	Prospective Observational	Index MI	463	79 (17.1)	98.7% (93.2–100%)	99.0% (94.3–100%)
Bandstein et al. (2014) [30]	Roche, Elecsys	<5 ng/L *	60.1	Retrospective Observational	MI at 30 days	14,612	788 (5.4)	Not Reported	99.8% (99.7–99.9%)
Gilje et al. (2024) [37]	Roche, Elecsys	<9 ng/L	66.5	Retrospective Observational	MI or Death at 30 days	132,021	7668 (5.8)	95.8% (95.4–96.3%)	99.6% (99.6–99.7%)
	Roche, Elecsys	<9 ng/L	57.3	Prospective Observational	MI or Death at 30 days	1167	88 (7.7)	95.7% (89.4–98.8%)	99.4% (98.5–99.8%)
Johannessen et al. (2021) [31]	Roche, Elecsys	<5 ng/L	33.3	Prospective Observational	Index MI	1711	61 (3.6)	100% (94.1–100%)	100% (99.4–100%)
Khand et al. (2017) [32]	Roche, Elecsys	<5 ng/L *	40.7	Prospective Observational	MACE at 6 weeks	1642	211 (12.9)	99.10%	99.7% (98.9–100%)
Body et al. (2016) [33]	Roche, Elecsys	<5 ng/L	43.7	Prospective Observational	Index MI	1282	213 (16.6)	98.1% (95.3–99.5%)	99.3% (98.2–99.8%)
McRae et al. (2019) [38]	Roche, Elecsys	<6 ng/L	42.6	Prospective Observational	MI at 7 days	7130	411 (5.8)	99.8% (98.4–100%)	N/A
High-Sensitivity Cardiac Troponin I									
Hickling et al. (2024) [23]	Abbott, Architect	<2 ng/L	49.5	Retrospective Observational	CV death and MI at 30 days	6633	16 (0.25)	100%	100%
Shah et al. (2015) [28]	Abbott, Architect	<5 ng/L	47.5	Prospective, observational	CV death and MI at 30 days (inc of index event)	4870	921 (18.9%)	NR	99.6%
Fabre-Estremera et al. (2023) [34]	Siemens, Atellica	<10 ng/L	44.3	Prospective Observational	MI or Death at 30 days	1171	148 (12.6)	93.2% (87.9–96.7%)	98.1% (96.5–99.1%)
Body et al. (2020) [35]	Siemens, Centaur	<5 ng/L	50.4	Prospective Observational	Index MI	999	131 (13.1)	99.2% (95.8–100%)	99.8% (98.6–100%)
Sandoval et al. (2017) [24]	Abbott, Architect	<2 ng/L	27	Prospective Observational	Index MI	1631	170 (10.4)	98.8% (98.2–99.6%	99.6% (98.9–100%)
		<5 ng/L	50					94.7% (91.3–98.1%)	98.9% (98.2–99.6%)
Apple et al. (2022) [39]	Siemens, VTLi	<4 ng/L	17.8	Prospective Observational	Index MI	1486	81 (5.5)	98.8% (93.3–100%)	99.8% (99.1–100%)
Sandoval et al. (2019) [40]	Siemens, Atellica	<2 ng/L	23	Prospective Observational	MI or Death at 30 days	2212	277 (12.5)	99.3% (98.3–100%)	99.6% (98.9–100%)
		<3 ng/L	33					98.6% (97.2–100%)	99.5% (98.9–100%)
	Siemens, Centaur	<2 ng/L	21					99.6% (98.9–100%)	99.8% (99.3–100%)
		<3 ng/L	31					98.9% (97.7–100%)	99.6% (99.1–100%)
Greenslade et al. (2018) [41]	Beckman Coulter, Access	<2 ng/L	34.1	Retrospective Observational	Index MI	1871	98 (5.2)	99.0% (94.4–100%)	99.8% (99.1–100%)

* Additionally required a non-ischaemic ECG to fulfil SSRO. Abbreviations: CI indicates confidence interval; CV, cardiovascular; ECG, electrocardiogram; MACE, major adverse cardiac events; MI, myocardial infarction and SSRO, single sample rule-out.

## 4. Single Sample Rule-In Using ADPs

The purpose of single sample rule-in (SSRI) is to triage high-risk patients to rapidly receive appropriate monitoring, investigations and evidence-based treatments [9]. Those who do not fulfil SSRI have serial hs-cTn sampling to determine the triage recommendation from the ADP. Therefore, the optimal SSRI threshold should prioritise positive predictive value (PPV), ensuring that patients selected for early management have a high likelihood of MI.

The ESC has published assay-specific SSRI thresholds in their most recent guidelines [22]. These were derived from observational studies, the majority of which are from the Advantageous Predictors of Acute Coronary Syndrome Evaluation cohort in Switzerland [42,43,44,45]. In these studies, the PPV of SSRI was consistently >70% for both hs-cTnT and hs-cTnI [42,43,44,45].

However, age and renal dysfunction are important confounding factors that can affect the performance of SSRI. These populations are more likely to have chronic myocardial injury and elevated baseline troponin levels, making it often impossible to distinguish acute from chronic injury using a single measurement. In such cases, serial sampling is required to confirm the dynamic change of acute myocardial injury. However, other causes of acute myocardial injury to type 1 MI, namely type 2 MI and acute non-ischaemic myocardial injury, both of which can be a result of tachyarrhythmias, sepsis, acute heart failure and pulmonary embolism, are more common in the elderly and those with chronic kidney disease. This should reduce the specificity and PPV for type 1 MI in these populations.

Despite this, observational studies have reported similar or even higher PPV in older patients and those with renal impairment. This likely reflects a higher underlying prevalence of MI in these groups, rather than improved diagnostic accuracy of the assay itself [42,43].

Therefore, whilst the data is extensive, further validation of these thresholds in cohorts with different demographics and ethnicities would be of benefit. The performance of the assay recommended thresholds in older or more co-morbid populations is less well established.

## 5. The European Society of Cardiology Accelerated Diagnostic Pathways

With the advent of hs-cTns, in 2015 the ESC published its first ADP to assist in the assessment of patients with suspected MI [9]. This used serial values taken over three hours to rule in or rule out MI, and it aimed to shorten stay and standardise care.

To achieve rule-out, a patient had to have an acceptable hs-cTn value combined with a low risk symptomatology and global registry of acute coronary events (GRACE) score. Therefore, it used both biochemical and clinical factors like earlier diagnostic pathways that utilised contemporary troponin assays over a two-hour interval [15]. This flow-chart-like algorithm was simple to follow and provided clearer guidance for clinicians. However, its application remained dependent on local implementation. Laboratories were required to perform assay-specific quality control and determine their own upper reference limits (URLs), while individual centres also needed to define appropriate delta thresholds for serial measurements (where ‘delta’ refers to the change in troponin concentration between consecutive measurements). As a result, variability in practice persisted despite the introduction of this structured approach.

In the same guidelines the ESC also published an ADP with serial sampling over one hour [9]. Its publication was based on two main principles: firstly, hs-cTn is interpreted as a quantitative variable, even below the 99th percentile, and numerically very low values predicted no subsequent rise in troponin. Secondly, due to the linear early kinetics of cTn release post-infarction, changes in concentration over one hour could be used as a surrogate marker for changes over three or six hours [21]. At the time of guideline publication only three assays had been validated for the 0/1 h pathway: hs-cTnT (Roche, Elecsys) and two hs-cTnI assays (Abbott Architect and Siemens Dimensions Vista). In addition, these assay-specific ADPs had been both derived and validated in only two observational cohorts [20,21].

**Figure 2:**
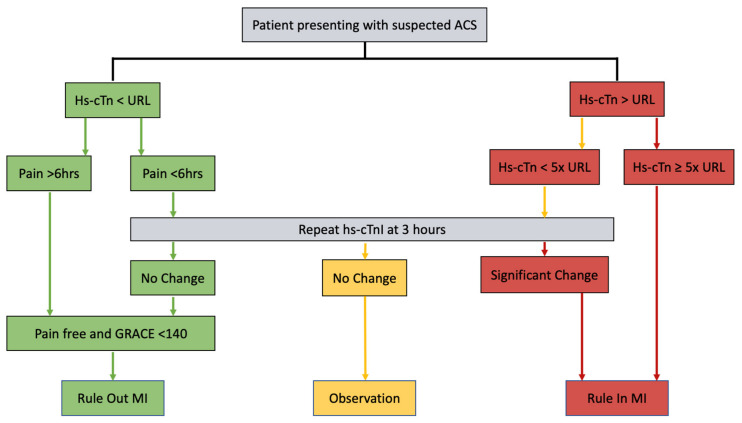
ESC 0/3 h ADP adapted from the 2015 guidelines on the management of patients presenting with suspected NSTEMI [9].

**Figure 3:**
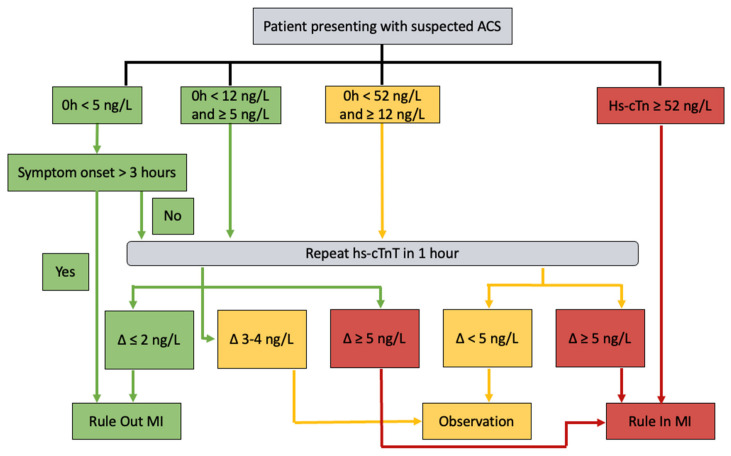
ESC 0/1 h ADP using hs-cTnT. Clinical decision limits are different for alternate assays, but the design remains the same [9].

The ESC 0–3 and 0–1 ADP concepts are shown in Figure 2 and Figure 3, respectively.

## 6. The ESC 0/3 h ADP

The original 0/3 h ADP uses the assay’s URL (upper reference limit) to triage patients as either high or low risk. This contrasts with the high-STEACS and ESC 0/1 h ADP, where a lower value is used near the limit of detection (LOD) as a predictive tool to triage patients to initially high or low risk for acute MI [9,28]. In addition, the ESC 0/3 h pathway utilises a GRACE score of <140 as an additional safety check prior to rule-out and discharge [9,46]. In contrast, the GRACE score is not used on the ESC 0/1 h ADP.

The 0/3 h ADP is a very frequent means of triaging acute chest pain worldwide [47]. The clinical and operational impact of implementing the ESC 0/3 h ADP instead of a conventional pathway has been assessed in observational studies [47,48]. One study of 10,315 consecutive patients demonstrated, when compared to conventional serial sampling over 6–12 h, that the ESC 0/3 h ADP was able to increase discharge rates and reduce length of stay in the ED, with no difference in rates of MI or cardiovascular death at 30 days or 1 year [48]. The strength of these findings provided the basis for wider adoption.

A summary of the current evidence base for the performance for both rule-out and rule-in of MI using the ESC 0/3 h ADP is illustrated in Table 4. Only one study published a sensitivity of rule-out of >97% [49]. No study was able to publish a sensitivity of over 99%, although the NPV was frequently greater than 99%.

To be classified as low risk and suitable for rule-out, patients must have acceptable hs-cTn levels, be pain-free, and have a GRACE score < 140. The clinical components of this assessment, particularly symptom resolution and GRACE scoring, are susceptible to reporting bias, especially in retrospective observational studies. As a result, patients may be incorrectly classified as low risk, leading to an overestimation of those eligible for discharge and potentially distorting estimates of safety, including sensitivity and rates of missed events.

Overall, observational studies suggest that the ESC 0/3 h pathway may have suboptimal sensitivity for both index MI and 30-day major adverse cardiac events (MACE), with only one study in Table 4 reporting a rule-out sensitivity greater than 95%. However, these findings should be interpreted with caution, as observational data are subject to reporting bias, which may lead to underestimation of the true sensitivity of the pathway. Further prospective implementation studies evaluating clinician decision-making are required to more accurately determine its real-world safety.

## 7. The ESC 0/1 h ADP

The ESC 0/1 h ADP has been subject to validation in multiple observational studies, one RCT and one meta-analysis [20,21,44,45,49,50,55,56,57,58,59,60,61,62,63,64]. Except for the difference in delta times, the other point of departure between the ESC 0/1 h and the conventional 0/3 h ADP is the value of the clinical decision limit used for rule-out.

A summary of the clinical performance of the ESC 0/1 h ADP for both rule-out and rule-in is presented in Table 5. Apart from one small observational study of 406 patients [50], reported rule-out sensitivity exceeds 96% across studies and is greater than 98% in most [17,20,44,45,49,55,56,57,58,59,60,61,63,64].

In a recent meta-analysis by Chiang et al., the pooled sensitivity for rule-out was 99.1% (95% CI 98.5–99.5%) [62], with six included studies reporting a sensitivity of 100%. Although the majority of included studies are observational, they encompass diverse populations from Western Europe, China, Australasia, and the United States, supporting the generalisability of these findings.

The only RCT available that assesses the performance of the ESC 0/1 h when implemented into clinical practice reported a sensitivity of 96.6% (95% CI 88.3–99.6%) for those who fulfilled rule-out [63]. This trial did not include the index MI presentation and only re-presentation MIs as the primary endpoint. Therefore, only events after discharge from initial presentation were included, up to 30 days. There were only 10 re-presentation type 1 MIs in the entire population, and one of these occurred in those discharged by clinicians using the ESC 0/1 h pathway (sensitivity 90%). Total event numbers are low, but further trials are required that assess the ESC 0/1 h ADP clinical performance when used in the real world.

The observation zone of the ESC 0/1 h pathway helps preserve safe and effective rule-out whilst ensuring the rule-in zone remains efficient. However, this observation zone can represent a diagnostic challenge for clinicians in the real world. The current guidelines endorse a repeat three-hour hs-cTn sample with the potential addition of either an anatomical (CT coronary angiography) or functional (stress echocardiography, MRI or a myocardial perfusion scan) test to exclude MI and significant coronary artery disease in this cohort.

Apart from one observational study from China by Dongxu et al., rule-in specificity exceeded 82% in all studies and 94% in the majority. However, PPV varied considerably, ranging from just under 40% to nearly 90%. In the meta-analysis, pooled specificity and PPV were 94.0% (95% CI 90.7–96.2%) and 65.1% (95% CI 56.3–73.0%), respectively [62].

These findings suggest relatively strong rule-in performance, which may be higher than expected given the lower clinical decision limits (CDLs) used in the ESC 0/1 h pathway. While such thresholds improve sensitivity and reduce missed MIs, they are also likely to increase the number of patients with chronic or non-ischaemic myocardial injury meeting rule-in criteria. Consequently, a reduction in specificity would be anticipated. This expected trade-off is supported by the previous literature, where conventional troponin assays typically demonstrate higher specificity and PPV than hs-cTn assays [10,65].

Evaluation of the implementation of the ESC 0/1 h ADP remains limited. Two prospective observational studies have been published: one conducted in a single chest pain unit in Germany and another two-centre study in Switzerland and Argentina.

The German cohort study demonstrated a reduction in patient length of stay by just over two hours when using the ESC 0/1 h pathway compared with the 0/3 h ADP [49]. In contrast, the Switzerland–Argentina study, which lacked a comparator group, reported a mean length of stay of 2.5 h, shorter than the 0/3 h sampling interval [56].

However, the generalisability of these findings is limited. The German study was conducted in a specialised chest pain unit staffed by cardiology clinicians, a setting likely associated with fewer operational delays than a typical emergency department. Furthermore, healthcare systems in Switzerland and Argentina differ from publicly funded systems such as the UK.

Overall, the ESC 0/1 h pathway demonstrates strong rule-out performance when its CDLs are applied retrospectively to patient populations. However, evidence evaluating its real-world implementation remains limited. Only one randomised controlled trial has assessed clinician decision-making using this pathway, reporting a discharge sensitivity of 90%, although event numbers were low [63].

The ESC 0/1 h ADP is currently recommended in the most recent guidelines over the existing 0/3 h ADP [22]. The current observational evidence does concur with this endorsement. However, further studies are required to evaluate the effectiveness and feasibility of the ESC 0/1 h pathway in real-world environments, particularly in publicly funded healthcare systems such as the UK, where operational pressures may impact implementation [66].

## 8. The Optimal Sampling Delta

Based on troponin release kinetics, a longer sampling interval may be expected to improve diagnostic performance. As troponin concentrations rise over time following MI, a three-hour interval is likely to produce a larger change in concentration (delta), which may facilitate differentiation between acute and chronic myocardial injury [21,67].

In contrast, the ESC 0/1 h pathway relies on smaller absolute changes in troponin levels, often at concentrations near or below the upper reference limit, where analytical variability is greater. This should make the risk of misclassification higher using a delta over one rather than three hours. This would be particularly relevant in patients with low-level troponin elevations [68].

**Figure 4:**
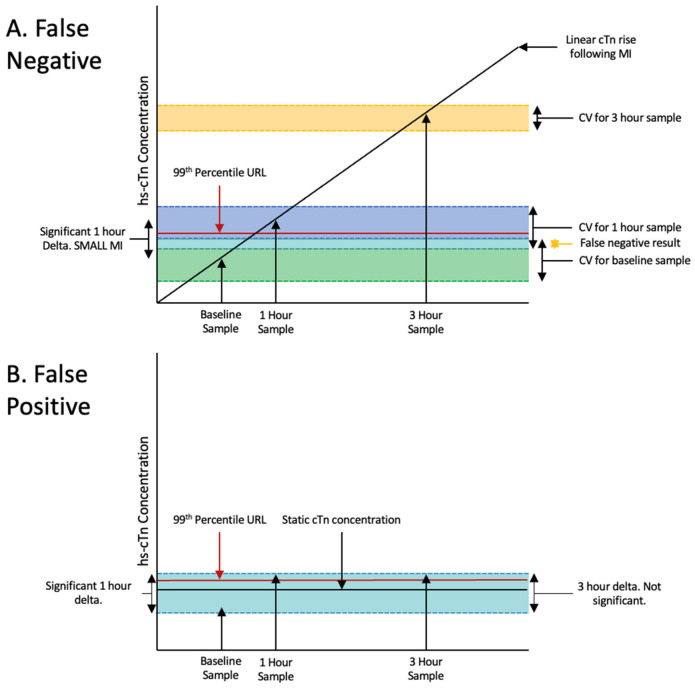
Graphical representation of the theoretical concern for using a one-hour delta at low hs-cTn concentrations. It demonstrates the mechanism by which an overlap in CV between two different results from a small MI can give the appearance of no delta, resulting in a false negative. (**B**) Results from the lowest possible value due to assay CV and then one from the highest could mimic the small delta seen in the 1-h ADP, resulting in a false positive. This change would still be deemed non-significant using a 3-h delta. Hs-cTn indicates high-sensitivity cardiac troponin; MI, myocardial infarction and CV, coefficient of variation.

However, the extent to which this theoretical limitation impacts clinical performance remains uncertain. A recent study has suggested than analytical variability may need to be greater than current performance specifications allow in order to be clinically significant [68]. Figure 4 is a graphical illustration of why analytical variability is a theoretically greater challenge for one-hour rather than three-hour sampling.

Despite the theoretical advantages of the 0/3 h ADP, subtle differences in the design of the ESC 0/1 and 0/3 h ADPs appear to impact its rule-out performance. Firstly, the 0/3 h algorithm uses the URL of the assay to triage to rule-out or not. This contrasts with the 0/1 h, which uses an assay-specific rule-out clinical decision limit lower than the assay URL. Secondly, the 0/1 h algorithm has a clearly defined safety net—the observation zone. This incorporates patients with elevated hs-cTn concentrations that do not fulfil rule-in criteria. Thirdly, as mentioned previously, the 0/3 h algorithm utilises clinical features as well as hs-cTn concentrations to triage patients. This can be difficult to quantify in observational studies, and these additional parameters, normal ECG and a GRACE score of <140, are often not quoted. In contrast, the ESC 0/1 h only uses hs-cTn concentrations, so it is easily extrapolated in observational studies and therefore less susceptible to this reporting bias. Therefore, observational comparisons of pathway performance are fraught with bias and potential error.

Despite the published data appearing to favour the ESC 0/1 h ADP, the ESC 0/3 h pathway continues to be popular [47]. This is likely due to the difficulties in operationalising the ESC 0/1 h pathways into busy EDs. Frequent repeated sampling will put additional strain on phlebotomy and clinical staff, and there is unease of repeat sampling at 1 h when the 0 h sampling may not be known at 1 h.

When the observational data presented in Table 4 and Table 5 are compared, it appears clear that the ESC 0/1 ADP outperforms the 0/3 ADP in the rule-out of MI. In the recent meta-analysis, the pooled sensitivity of the 0/1 and 0/3 ADPs was 99.1% (95% CI 98.5–99.5%) and 93.7% (95% CI 87.4–97.0%), respectively [62], with both ADPs maintaining similar specificity and PPV for rule-in.

However, only three studies have compared both ADPs using the same patient cohort, and these are shown in Table 6 [50,53,69]. The first study by Chapman et al. retrospectively analysed the performance of the ESC 0/3 and 0/1 h ADPs using a sub-study of patients recruited into the high-STEACS trial [28]. Unfortunately, the cohort used to assess the 0/1 ADP was only 21% of the size of the cohort used in the 0/3 h ADP and is therefore likely to be under-powered to detect differences in clinical performance.

All three studies using either hs-cTnT or TnI found that the ESC 0/1 h ADP was either superior or non-inferior to the 0/3 h pathway with regards to safety. However, none of these studies compared implementation of these ADPs directly, including their impacts on patient ED length of stay [50,53,69].

Despite the ESC 0/1 h ADP’s published safety and class 1(B) recommendation from the ESC, there remains no RCT that directly compares both the safety of clinician decision-making and impact on ED efficiency of the ESC 0/1 h ADP against its 0/3 h counterpart.

## 9. High-STEACS Accelerated Diagnostic Protocol

The High-STEACS ADP was developed from patients recruited into a cluster randomised controlled trial in Scotland, UK [28,50,70,71]. The study evaluated the introduction of a hs-cTnI assay with sex-specific upper reference limits, compared with contemporary troponin testing in patients with suspected acute coronary syndrome.

**Figure 5:**
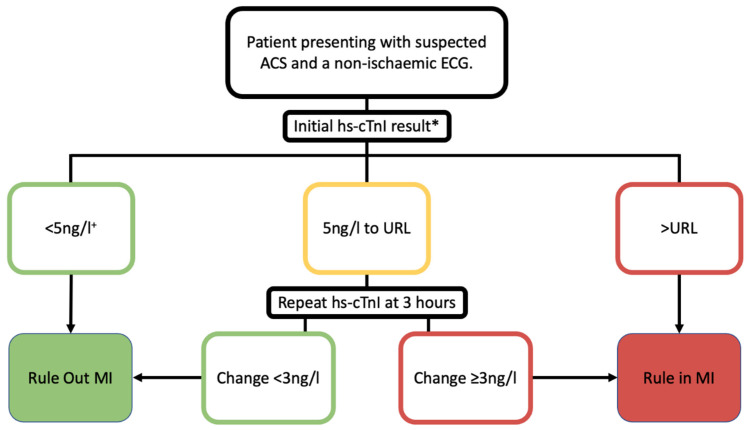
The high-STEACS ADP [72]. * Applicable to Abbott Architect and Siemens Atellica hs-cTnI assays. ^+^ Repeat hs-cTnI at 3-h if symptom onset was ≤2 h from blood draw. ACS indicates acute coronary syndrome; ECG, electrocardiogram; hs-cTnI, high-sensitivity cardiac troponin I; URL, upper reference limit and MI, myocardial infarction.

The pathway was initially developed using the Abbott Architect assay and subsequently validated with the Siemens Atellica hs-cTnI assay. It incorporates assay-specific CDLs and serial troponin changes over three hours to guide patient triage (Figure 5). Importantly, the pathway is intended for use only in patients with a non-ischaemic ECG.

An SSRO threshold of <5 ng/L, using the Abbott Architect hs-cTnI assay, in conjunction with non-ischaemic ECG was validated in a prospective cohort study of 4870 patients and reported an NPV of 99.6% (95% CI 99.3–99.8%) for rule-out of MI [28].

Three observational studies have compared the high-STEACS pathway with the ESC 0/3 h ADP using sex-specific assay CDLs (Table 7) [50,51,71]. Two studies utilised the Abbott Architect hs-cTnI assay, while one used the Siemens Atellica hs-cTnI assay. Across all studies, high-STEACS demonstrated superior safety for rule-out compared with the ESC 0/3 h pathway.

External validation of the high-STEACS pathway has been performed in two studies from the United States, one using hs-cTnI and the other hs-cTnT (Table 4) [73,74]. Both studies reported lower sensitivity for rule-out of cardiac death or MI (90% and 91%, respectively), with corresponding reductions in negative predictive value compared with findings from the original high-STEACS cohorts. These differences highlight potential limitations in generalisability and the need for further validation in diverse populations.

In high-STEACS, the CDL for the delta used to determine triage category is 3 ng/L for both the Abbott and Siemens hs-cTnI assays. In contrast, the ESC 0/3 h ADP uses higher delta thresholds, ranging from 8 to 17 ng/L (Abbott assay) and 17 to 26 ng/L (Siemens assay), with sex-specific differences. These thresholds are between 2.6 and 8.6 times higher than those used in high-STEACS [50,51,71].

Theoretically, a smaller delta would be expected to improve sensitivity at the expense of rule-in performance. However, published data suggest that rule-in performance appears comparable between the ESC 0/3 h and high-STEACS pathways (Table 7).

In all three major observational studies evaluating high-STEACS, 67–75% of those with elevated hs-cTnI levels were diagnosed with type 1 MI [50,51,71]. This represents an enriched study population with a higher prevalence of MI than would typically be seen in unselected patients presenting with suspected acute coronary syndrome.

Such enrichment is likely to overestimate diagnostic performance, particularly specificity and PPV. This is supported by external validation in a US cohort using hs-cTnT, where a greater proportion of patients had acute or chronic myocardial injury rather than infarction [73]. In this study of 1,351 patients, the PPV for 30-day cardiac death or MI was only 31.6%, which may more accurately reflect real-world rule-in performance.

In contrast to the most recent guidelines from the ESC, the high-STEACS ADP utilises sex-specific hs-cTn cut-offs to determine triage category [22,50,51,71]. There has been theoretical concern that use of an overall cut-off, used in both ESC ADPs, may result in false negatives in the female population. However, this has not been consistently demonstrated in the literature [22]. The high-STEACS ADP was designed with sex-specific cut-offs in order to potentially mitigate against these female false negatives. Clinical performance does appear comparable to ESC pathways using overall hs-cTn CDLs.

When compared to the ESC 0/3 h ADP, high-STEACS appears to have superior rule-out performance in the population in which it was initially derived and validated. However, both rule-out and rule-in was less impressive in external validation in US cohorts. Further external validation of the high-STEACS ADP is required to better understand if the high-STEACS ADP and its assay-specific thresholds are generalisable to different populations than those it was derived in.

## 10. Summary

This review summarises the evolution and current evidence base for ADPs in the assessment of suspected myocardial infarction. While rapid pathways such as the ESC 0/1 h and high-STEACS ADP demonstrate strong diagnostic performance, several practical challenges remain. These include delays in laboratory turnaround times, uncertainty regarding interpretation of intermediate ‘observation zone’ results, variability in optimal delta thresholds and incorporation of new technologies, such as point-of-care hs-cTn assays.

In addition, the relatively low PPV for rule-in across ADPs continues to limit their ability to confidently identify high-risk patients. Further refinement of troponin thresholds and delta values may improve patient stratification and support more efficient decision-making, including early discharge or targeted outpatient investigation.

## Figures and Tables

**Table 1:** Definitions of ‘sensitive’ and high-sensitivity cTn assays.

Assay Definition	Coefficient of Variation at the 99th Percentile Upper Reference Limit.	Proportion of Patients with Reportable Levels < 99th Percentile Upper Reference Limit.
Sensitive	<10%	20–50%
High-Sensitivity	<10%	>50%

**Table 4:** Grouped summary of current studies analysing the clinical performance of the ESC 0/3 h ADP in the diagnosis of MI.

Article	Study Design	Biomarker	Primary Outcome	Population (n)	Prevalence of MI n (%)	Rule-Out			Rule-In		
						n (%)	Sensitivity (95% CI)	NPV (95% CI)	n (%)	Specificity (95% CI)	PPV (95% CI)
Chapman et al. [50]	Retrospective Observational	hs-cTnI	MI or CV death at 30 days	1920	274 (14.3)	1248 (65)	90.8% (87.4–94.1%)	98% (97.1–98.7%)	672 (35)	74.4% (72.3–76.5%)	37.5% (33.9–41.2%)
Chapman et al. [51]	Prospective Observational	hs-cTnI	MI or CV death at 30 days	1886	271 (14.4)	1328 (70)	89.9% (86.3–93.4%)	97.9% (97.1–98.6%)	558 (30)	80.5% (78.6–82.5%)	43.7% (39.7–47.9%)
Pickering et al. [52]	Retrospective Observational	hs-cTnI	Index MI	1061	132 (12)	958 (90)	93.2% (87.5–96.8%)	N/A	103 (10)	98.2% (97.1–98.9%)	83.5% (74.9–90.1%)
		hs-cTnT		985	134 (14)	885 (90)	94.8% (89.5–97.9%)	N/A	100 (10)	96.7% (95.3–97.8%)	72.0% (62.1–80.5%)
Haller et al. [53]	Prospective Observational	hs-cTnI	Death, MI, revascularisation or cardiac hospitalisation	Without DM—2910	489 (16.8)	2190 (75)	93.8% (91.2–95.9%)	98.7% (98.2–99.1%)	662 (23)	90.2% (89.0–91.4%)	63.7% (60.0–67.4%)
		hs-cTnI		With DM—513	90 (17.6)	388 (66)	89.8% (82.9–94.6%)	96.4% (93.9–98.2%)	159 (31)	85.8% (82.0–89.1%)	64.8% (56.8–72.2%)
Sandoval et al. [54]	Prospective Observational	hs-cTnI	Death or MI at 30 days	211	N/A	206 (35)	94.4% (89.0–99.7%)	98.1% (96.2–99.9%)	N/A	N/A	N/A
Stoyanov et al. [49]	Prospective Observational	hs-cTnT	Index MI	948	241 (26)	550 (58)	97.1% (94.1–98.8%)	98.7% (97.4–99.5%)	398 (42)	76.8% (73.5–79.9%)	58.8% (53.8–63.7%)

MI indicates myocardial infarction; NPV, negative predictive value; PPV, positive predictive value; hs-cTnI, high-sensitivity cardiac troponin I; hs-cTnT, high-sensitivity cardiac troponin T and DM, diabetes mellitus.

**Table 5:** Grouped summary of current studies analysing the clinical performance of the ESC 0/1 h ADP in the diagnosis of MI.

Article	Study Design	Biomarker	Primary Outcome	Population	Prevalence of MI n (%)	Rule-Out			Rule-In		
						n (%)	Sensitivity (95% CI)	NPV (95% CI)	n (%)	Specificity (95% CI)	PPV (95% CI)
Rubini Gimenez et al. [20]	Prospective Observational	hs-cTnI	Index MI	906—Derivation	162 (18)	505 (56)	97.6% (93.8–99.3%)	99.2% (98.0–99.8%)	163 (18)	94.5% (92.6–96.0%)	74.9% (67.4–81.3%)
				905—Validation	167 (19)	457 (51)	98.8% (95.7–99.9%)	99.6% (98.4–100%)	172 (19)	93.8% (91.8–95.4%)	73.9% (66.7–80.2%)
Reichlin et al. [17]	Prospective Observational	hs-cTnT	Index MI	1320	229 (17)	786 (60)	99.6% (97.6–99.9%)	99.9% (99.3–100%)	216 (16)	95.7% (94.3–96.8%)	78.2% (72.1–83.6%)
Mueller et al. [55]	Prospective Observational	hs-cTnT	Index MI	1282	213 (17)	813 (63)	96.7% (93.3–98.7%)	99.1% (98.2–99.7%)	184 (14)	96.0% (94.7–97.2%)	77.2% (70.4–83.0%)
Boeddinghaus et al. [45]	Prospective Observational	hs-cTnI	Index MI	672—Derivation	Not Quoted	304 (45)	99.2% (95.6–100%)	99.7% (97.7–100%)	121 (18)	95.3% (93.1–96.9%)	78.5% (70.1–85.5%)
		hs-cTnI	Index MI	675—Validation	Not Quoted	313 (46)	99.1% (95.3–100%)	99.7% (97.8–100%)	120 (18)	94.1% (91.8–95.9%)	72.5% (63.6–80.3%)
Twerenbold et al. [44]	Prospective Observational	hs-cTnT	Index MI	4368	735 (17)	2493 (57)	99.3%	99.8%	768 (18)	94.6%	74.5%
		hs-cTnI		3500	588 (17)	1533 (44)	99.1%	99.7%	800 (23)	89.6%	62.3%
Twerenbold et al. [56]	Prospective Observational	hs-cTnT	CV death or MI at 30 days	2296	227 (10)	1420 (62)	99.1%	99.9%	295 (13)	95.2%	66.1%
Stoyanov et al. [49]	Prospective Observational	hs-cTnT	Index MI	1146	231 (20)	519 (45)	98.7% (96.3–99.7%)	99.4% (98.3–99.9%)	382 (33)	82.2% (79.6–84.6%)	57.3% (52.2–62.3%)
Pickering et al. [57]	Retrospective Observational	hs-cTnT	Index MI	2222	240 (11)	1426 (64)	97.1% (94.0–98.8%)	99.5% (99.0–99.8%)	292 (13)	94.6% (93.4–95.5%)	63.4% (57.5–68.9%)
		hs-cTnI		2222	240 (11)	1205 (54)	98.8% (96.4–99.7%)	99.8% (99.3–99.9%)	310 (14)	95.0% (94.0–95.9%)	68.1% (62.6–73.2%)
Amann et al. [58]	Prospective Observational	hs-cTnT	Index MI	1317	486 (37)	398 (30)	99.8% (98.5–100%)	99.8%	449 (34)	94.3% (92.6–95.8%)	89.5%
Andruchow et al. [64]	Prospective Observational	hs-cTnT	Index MI	350	37 (11)	219 (63)	97.3% (85.3–99.9%)	99.5%	37 (11)	96.5%	70.3%
Lehmacher et al. [59]	Retrospective Observational	hs-cTnI	Index MI	1879	257 (14)	837 (45)	97.7% (95.0–99.1%)	99.3% (98.4–99.7%)	382 (20)	88.0% (86.3–89.6%)	50.8% (45.7–55.9%)
Nowak et al. [60]	Prospective Observational	hs-cTnI	Index MI	2346	278 (12)	1065 (50)	98.7% (96.3–99.7%)	99.7% (96.3–99.7%)	265 (13)	95.7% (94.7–96.6%)	69.4% (63.5–74.9%)
Dongxu et al. [61]	Prospective Observational	hs-cTnT	Index MI	577	106 (18)	278 (48)	100%	100%	151 (26)	62.9% (58.5–67.2%)	37.1% (31.3–42.8%)
Chapman et al. [50]	Retrospective Observational	hs-cTnI	CV death or MI at 30 days	406	31 (8)	262 (65)	92.2% (83.0–99.4%)	99.0% (97.6–99.8%)	28 (7)	98.2% (96.8–99.4%)	77.6% (61.1–90.5%)
Chew et al. [63]	Randomised Controlled Trial	hs-cTnT	Death or MI in 30 days	1646	59 (4)	1187 (72)	96.6% (88.3–99.6%)	99.8% (99.4–100%)	136 (8)	94.7% (93.4–95.7%)	38.2% (30.0–47.0%)

MI indicates myocardial infarction; NPV, negative predictive value; PPV, positive predictive value; hs-cTnI, high-sensitivity cardiac troponin I and hs-cTnT, high-sensitivity cardiac troponin T.

**Table 6:** Observational studies directly comparing the ESC 0/1 and 0/3 h ADPs.

Article	hs-cTn Subtype	ADP	Population	Prevalence of MI n (%)	Rule-Out			Rule-In		
					n (%)	Sensitivity	NPV	n (%)	Specificity	PPV
Chapman et al. [50]	hs-cTnI	1 h	406	31 (8)	262 (65)	92.2% (83.0–99.4%)	99.0% (97.6–99.8%)	28 (7)	98.2% (96.8–99.4%)	77.6% (61.1–90.5%)
		3 h	1920	274 (14.3)	1248 (65)	90.8% (87.4–94.1%)	98.0% (97.1–98.7%)	672 (35)	74.4% (72.3–76.5%)	37.5% (33.9–41.2%)
Badertscher et al. [69]	hs-cTnT	1 h	2547	387 (15)	60%	N/A	99.8% (99.4–99.9%)	N/A	N/A	N/A
		3 h	2547	387 (15)	44%	N/A	99.7% (99.2–99.9%)	N/A	N/A	N/A
	Hs-cTnI	1 h	2197	327 (15)	52%	N/A	99.6% (99.1–99.9%)	N/A	N/A	N/A
		3 h	2197	327 (15)	51%	N/A	97.8% (96.7–98.5%)	N/A	N/A	N/A
Haller et al. [53]	hs-cTnI	1 h—No DM	1669	N/A	698 (42)	99.3% (97.4–99.9%)	99.7% (99.0–100%)	362 (22)	91.5% (89.9–92.9%)	67.4% (62.3–72.2%)
		3 h—No DM	2910	N/A	2190 (75)	93.8% (91.2–95.9%)	98.7% (98.2–99.1%)	662 (23)	90.2% (89.0–91.4%)	63.7% (60.0–67.4%)
		1 h—DM	251	N/A	56 (22)	98.4% (91.3–100%)	98.2% (90.4–100%)	74 (30)	84.7% (78.7–89.5%)	60.8% (48.8–72.0%)
		3 h—DM	513	N/A	338 (66)	89.8% (82.9–94.6%)	96.4% (93.9–98.2%)	159 (31)	85.8% (82.0–89.1%)	64.8% (56.8–72.2%)

Hs-cTn indicates high-sensitivity cardiac troponin; MI, myocardial infarction; NPV, negative predictive value and PPV, positive predictive value.

**Table 7:** Studies assessing the performance of the high-STEACS ADP or comparing it to the ESC 0/3 h ADP.

Article	hs-cTn Assay	ADP	Population	Prevalence of MI n (%)	Rule-Out			Rule-In		
					n (%)	Sensitivity	NPV	n (%)	Specificity	PPV
Chapman et al. [71]	Abbott Architect hs-cTnI	ESC 3 h	1218	189 (16)	961 (79)	89.3% (84.9–93.5%)	97.9% (96.9–98.7%)	N/A	91.6% (89.9–93.3%)	66.5% (60.6–72.1%)
		High-STEACS	1218	189 (16)	904 (74)	97.7% (95.5–99.5%)	99.5% (99.0–99.9%)	N/A	87.6% (85.6–89.6%)	59.5% (54.1–64.9%)
Chapman et al. [50]	Siemens Atellica hs-cTnI	ESC 3 h	1920	274 (14)	1248 (65)	90.8% (87.4–94.1%)	98.0% (97.1–98.7%)	672 (35)	74.4% (72.3–76.5%)	37.5% (33.9–41.2%)
		High-STEACS	1920	274 (14)	1218 (63)	98.0% (96.4–99.5%)	99.5% (99.1–99.8%)	702 (37)	73.8% (71.7–75.9%)	38.8% (35.2–42.4%)
Chapman et al. [51]	Abbott Architect hs-cTnI	ESC 3 h	1886	273 (14)	1328 (70)	89.9% (86.3–93.4%)	97.9% (97.1–98.6%)	558 (30)	80.5% (78.6–82.5%)	43.7% (39.7–47.9%)
		High-STEACS	1917	273 (14)	1244 (65)	98.7% (97.4–99.8%)	99.7% (99.4–99.9%)	673 (35)	75.6% (73.5–77.7%)	40.6% (36.9–44.3%)
Ashburn et al. [73]	Roche cobas hs-cTnT	High-STEACS	1351	166 (12)	857 (63)	94.0% (89.2–97.1%)	98.8% (97.9–99.4%)	494 (37)	71.5% (68.8–74.0%)	31.6% (27.5–35.9%)
Sandoval et al. [74]	Abbott hs-cTnI	High-STEACS	1631	170 (10)	1256 (77)	90.3% (84.8–95.7%)	98.9% (98.3–99.6%)	-	-	-

ADP indicates accelerated diagnostic pathway; hs-cTnI, high-sensitivity cardiac troponin I; MI, myocardial infarction; NPV; negative predictive value and PPV, positive predictive value.

## Data Availability

No new data were created or analysed in this study. Data sharing is not applicable.
